# Supplementary material for: Hydrolytic Degradation of Polylactic Acid Fibers as a Function of pH and Exposure Time
Source: Molecules. 2021 Dec 13;26(24):7554. doi: 10.3390/molecules26247554 (PMC8706057; doi:10.3390/molecules26247554)
Supplement: Supplementary file 1 [file molecules-26-07554-s001.zip › molecules-1466933-SI.pdf]

molecules

MDPI

Article

Hydrolytic Degradation of Polylactic Acid Fibers as a Function of pH and Exposure Time

Radhika Vaid [1], Erol Yildirim [2], Melissa A. Pasquinelli [1,3] and Martin W. King [1,4,*]

[1] Fiber and Polymer Science Program, Wilson College of Textiles, NC State University, Raleigh, NC 27606, USA; rvaid@ncsu.edu (R.V.); melissa_pasquinelli@ncsu.edu (M.A.P.)
[2] Department of Chemistry, Middle East Technical University, 06800 Ankara, Turkey; erolyil@metu.edu.tr
[3] Department of Forest Biomaterials, College of Natural Resources, NC State University, Raleigh, NC 27606, USA
[4] College of Textiles, Donghua University, Shanghai 201620, China
* Correspondence: mwking2@ncsu.edu; Tel.: +1-919-291-2563

Appendix A: XRD Analysis

Table S1. Peak height and area ratios with respect to highest intensity peak measured from XRD.

pH / Peak Ratios		10	7.4	3	2	Control
**16.4:28.5**	*Peak Height Ratio*	70.15	65.13	70.96	68.11	-
**16.4:28.5**	*Area Under Curve Ratio*	150.28	155.63	187.21	223.68	-

Citation: Vaid, R.; Yildirim, E.; Pasquinelli, M.A.; King, M.W. Hydrolytic Degradation of Polylactic Acid Fibers as a Function of pH and Exposure Time. *Molecules* **2021**, *26*, 7554. https://doi.org/10.3390/molecules26247554

Academic Editor: Mihai Brebu

Received: 1 November 2021
Accepted: 19 November 2021
Published: 13 December 2021

Publisher's Note: MDPI stays neutral with regard to jurisdictional claims in published maps and institutional affiliations.

Copyright: © 2021 by the authors. Submitted for possible open access publication under the terms and conditions of the Creative Commons Attribution (CC BY) license (http://creativecommons.org/licenses/by/4.0/).

Appendix B: Fukui Analysis

Table S2: Fukui analysis illustrating charge distribution for the atoms in PLA chain, where Figures S2, S3 and S4 illustrate a pictorial representation for nucleophilic, electrophilic and radical addition, respectively.

Atom	Atom Type	f(nuc)	f(elec)	f(radikal)
1	C	0.00042	0.00209	0.001255
2	O	0.03854	0.03916	0.03885
3	C	0.05058	0.00185	0.026215
4	O	0.01693	0.01132	0.014125
5	H	0.0125	0.00655	0.009525
6	C	-0.00045	-0.00115	-0.0008
7	H	0.00501	0.00426	0.004635
8	H	0.0051	0.0046	0.00485
9	H	0.01165	0.00458	0.008115
10	H	0.00715	0.00367	0.00541
11	C	0.00215	0.00475	0.00345
12	O	0.03347	0.0716	0.052535
13	C	0.03587	0.00156	0.018715
14	O	0.01304	0.01859	0.015815
15	H	0.02395	0.01147	0.01771
16	C	-0.00312	-0.00147	-0.00229
17	H	0.00729	0.00676	0.007025
18	H	0.00712	0.0066	0.00686
19	H	0.01135	0.00749	0.00942
20	C	-0.00012	0.00656	0.00322
21	O	0.06375	0.09287	0.07831
22	C	0.07963	0.00125	0.04044

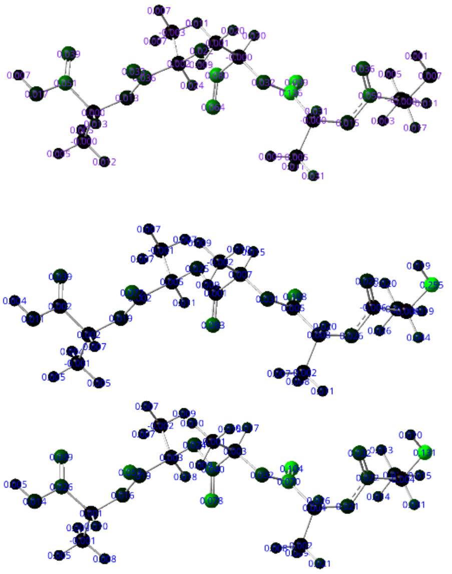

Figure S1: Nucleophilic, electrophilic and radical attack sites for PLA pentamer. High reactivity sites are shown by green color.

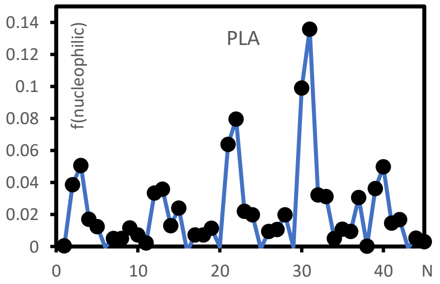

Figure S2: Fukui reactivity indices for nucleophilic addition

23	O	0.02201	0.02508	0.023545
24	H	0.01978	0.015	0.01739
25	C	-0.00132	-0.00158	-0.00145
26	H	0.0094	0.00878	0.00909
27	H	0.01069	0.00857	0.00963
28	H	0.01988	0.00963	0.014755
29	C	-0.00038	0.00801	0.003815
30	O	0.09893	0.10848	0.103705
31	C	**0.13587**	0.00469	0.07028
32	O	0.03216	0.03092	0.03154
33	H	0.03113	0.02042	0.025775
34	C	0.00504	-0.00169	0.001675
35	H	0.0109	0.00769	0.009295
36	H	0.0094	0.00704	0.00822
37	H	0.03067	0.01122	0.020945
38	C	0.00013	-0.0083	-0.00409
39	O	0.03619	0.02773	0.03196
40	C	0.04972	-0.00615	0.021785
41	O	0.01469	0.02642	0.020555
42	H	0.01689	0.0442	0.030545
43	C	-0.00076	0.01088	0.00506
44	H	0.00522	0.01986	0.01254
45	H	0.00307	0.02583	0.01445
46	H	0.01105	0.01852	0.014785
47	O	0.00677	0.25466	0.130715

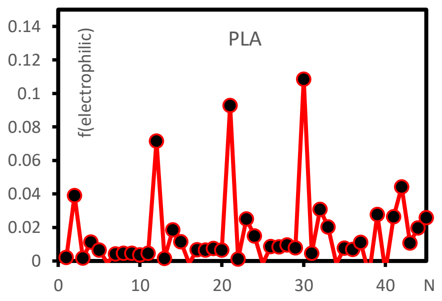

Figure S3: Fukui reactivity indices for electrophillic addition

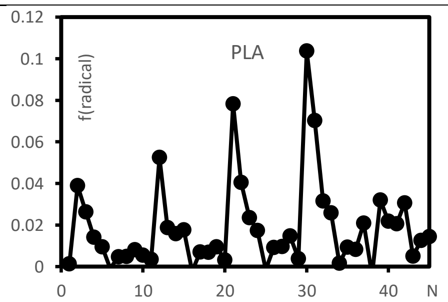

Figure S4: Fukui reactivity indices for radical addition

48	H	0.00108	0.01909	0.010085	

References

1. Barrows, T. Degradable implant materials: A review of synthetic absorbable polymers and their applications. *Clin. Mater.* **1986**, *1*, 233–257, https://doi.org/10.1016/s0267-6605(86)80015-4.
2. Gomes, M.; Reis, R.L. Biodegradable polymers and composites in biomedical applications: From catgut to tissue engineering. Part 1 Available systems and their properties. *Int. Mater. Rev.* **2004**, *49*, 261–273, https://doi.org/10.1179/095066004225021918.
3. Papazoglou, L.G.; Tsioli, V.; Papaioannou, N.; Georgiadis, M.; Savvas, I.; Prassinos, N.; Zavros, N. Comparison of absorbable and nonabsorbable sutures for intradermal skin closure in cats. *Can. Vet. J.* **2010**, *51*, 770.
4. Sajid, M.S.; McFall, M.R.; Whitehouse, P.A.; Sains, P.S. Systematic review of absorbable vs non-absorbable sutures used for the closure of surgical incisions. *World J. Gastrointest. Surg.* **2014**, *6*, 241.
5. Luckachan, G.E.; Pillai, C.K.S. Biodegradable Polymers- A Review on Recent Trends and Emerging Perspectives. *J. Polym. Environ.* **2011**, *19*, 637–676, https://doi.org/10.1007/s10924-011-0317-1.
6. Suzuki, S.; Ikaka, Y. *Sutures for Wound Closure. Biomaterials for Surgical Operation Electronic Resource, 189–196*; Humana Press: Totowa, NJ, USA, Springer: Berlin/Heidelberg, Germany, 2012.
7. Chen, G.-Q.; Wu, Q. The application of polyhydroxyalkanoates as tissue engineering materials. *Biomaterials* **2005**, *26*, 6565–6578, https://doi.org/10.1016/j.biomaterials.2005.04.036.
8. Williams, S.F.; Rizk, S.; Martin, D.P. Poly-4-hydroxybutyrate (P4HB): A new generation of resorbable medical devices for tissue repair and regeneration. *Biomed. Eng.* **2013**, *58*, 1–13.
9. Nair, N.; Laurencin, C.T. Biodegradable polymers as biomaterials. *Prog. Polym. Sci.* **2007**, *32*, 762–798.
10. Gajjar, C.R.; King, M.W. *Resorbable Fiber-Forming Polymers for Biotextile Applications*, 1st ed.; Springer: Berlin/Heidelberg, Germany, 2014.
11. Vroman, I.; Tighzert, L. Biodegradable polymers. *Materials* **2009**, *2*, 307–344.
12. Ulery, B.D.; Nair, L.S.; Laurencin, C.T. Biomedical applications of biodegradable polymers. *J. Polym. Sci. B Polym. Phys.* **2011**, *49*, 832–864.
13. King, M.W.; Gupta, B.S.; Guidoin, R. (Eds.) *Biotextiles as Medical Implants*; Woodhead Publishing: Cambridge, UK, 2014.
14. Schlick, T. *Molecular Modeling and Simulation: An Interdisciplinary Guide*; Springer: New York, NY, USA, 2002.
15. Hinchliffe, A. *Molecular Modelling for Beginners,* 1. Aufl. ed.; Wiley: Hoboken, NJ, USA, 2005.
16. Astuti, A.D.; Mutiara, A.B. Performance Analysis on Molecular Dynamics Simulation of Protein Using GROMACS. *arXiv* **2009** arXiv:0912.0893.
17. Glotzer, S.C.; Paul, W. Molecular and Mesoscale Simulation Methods for Polymer Materials. *Annu. Rev. Mater. Res.* **2002**, *32*, 401–436, https://doi.org/10.1146/annurev.matsci.32.010802.112213.
18. Deshmukh, S.A.; Hanson, B.J.; Jiang, Q.; Pasquinelli, M.A. Perspectives on the Use of Molecular Dynamics Simulations to Characterize Filler-Matrix Adhesion and Nanocomposite Mechanical Properties. *Interface/Interphase in Polymer Nanocomposites*; John Wiley & Sons, Inc: Hoboken, NJ, USA, 2016; pp. 379–411.
19. Ishimoto, T.; Koyama, M. A review of molecular-level mechanism of membrane degradation in the polymer electro-lyte fuel cell. *Membranes* **2012**, *2*, 395–414.
20. Yu, T.H.; Sha, Y.; Liu, W.-G.; Merinov, B.V.; Shirvanian, P.; Goddard, W.A. Mechanism for Degradation of Nafion in PEM Fuel Cells from Quantum Mechanics Calculations. *J. Am. Chem. Soc.* **2011**, *133*, 19857–19863, https://doi.org/10.1021/ja2074642.
21. Smith, K.D.; Stoliarov, S.I.; Nyden, M.R.; Westmoreland, P.R. RMDff: A smoothly transitioning, forcefield-based representation of kinetics for reactive molecular dynamics simulations. *Mol. Simul.* **2007**, *33*, 361–368, https://doi.org/10.1080/08927020601156392.
22. Liang, T.; Devine, B.; Phillpot, S.R.; Sinnott, S.B. Variable Charge Reactive Potential for Hydrocarbons to Simulate Organic-Copper Interactions. *J. Phys. Chem. A* **2012**, *116*, 7976–7991, https://doi.org/10.1021/jp212083t.
23. Van Duin, A.C.T.; Dasgupta, S.; Lorant, F.; Goddard, W.A. ReaxFF: A Reactive Force Field for Hydrocarbons. *J. Phys. Chem. A* **2001**, *105*, 9396–9409, doi:10.1021/jp004368u.
24. Chenoweth, K.; Cheung, S.; van Duin, A.C.T.; Goddard, W.A.; Kober, E.M. Simulations on the thermal decomposition of a poly(dimethylsiloxane) polymer using the ReaxFF reactive force field. *J. Am. Chem. Soc.* **2005**, *127*, 7192–7202.
25. Chenoweth, K.; van Duin, A.C.T.; Goddard, W.A. ReaxFF reactive force field for molecular dynamics simula-tions of hydrocarbon oxidation. *J. Phys. Chem. A* **2008**, *112*, 1040.
26. van Duin, A.C.T.; Merinov, B.V.; Jang, S.S.; Goddard, W.A. ReaxFF Reactive Force Field for Solid Oxide Fuel Cell Systems with Application to Oxygen Ion Transport in Yttria-Stabilized Zirconia. *J. Phys. Chem. A* **2008**, *112*, 3133–3140, https://doi.org/10.1021/jp076775c.
27. Chenoweth, K.; van Duin, A.C.T.; Persson, P.; Cheng, M.; Oxgaard, J.; Goddard, W.A. Development and appli-cation of a ReaxFF reactive force field for oxidative dehydrogenation on vanadium oxide catalysts. *J. Phys. Chem. C* **2008**, *112*, 14645.
28. Sanz-Navarro, C.F.; Åstrand, P.-O.; Chen, D.; Rønning, M.; van Duin, A.C.T.; Jacob, T.; Goddard, W.A. Molecular Dynamics Simulations of the Interactions between Platinum Clusters and Carbon Platelets. *J. Phys. Chem. A* **2008**, *112*, 1392–1402, https://doi.org/10.1021/jp074806y.

29. Ojwang, J.G.O.; Van Santen, R.; Kramer, G.J.; Van Duin, A.C.; Goddard III, W.A. Modeling the sorption dynamics of NaH using a reactive force field. *J. Chem. Phys.* **2008**, *128*, 9.
30. Karatrantos, A.; Clarke, N.; Kröger, M. Modeling of polymer structure and conformations in polymer nanocompo-sites from atomistic to mesoscale: A review. *Polym. Rev.* **2016**, *56*, 385–428.
31. Chen, X.; Yuan, C.A.; Wong, C.K.; Ye, H.; Leung, S.Y.; Zhang, G. Molecular modeling of protonic acid doping of emeraldine base polyaniline for chemical sensors. *Sens. Actuators B Chem.* **2012**, *174*, 210–216, https://doi.org/10.1016/j.snb.2012.08.042.
32. Gajjar, C.R.; Stallrich, J.W.; Pasquinelli, M.A.; King, M.W. *ACS Omega* **2021**, *6 (24), 15920–15928*, doi: 10.1021/acsomega.1c01557.
33. Origin(Pro), Version 2019b, OriginLab Corporation, Northampton, MA, USA, https://www.originlab.com/2019b.
34. Scienomics MAPS version 4.2.0., www.scienomics.com. (Accessed on 26 May 2017).
35. Aktulga, H. M.; Fogarty, J. C.; Pandit, S. A.; Grama, A. Y. Parallel reactive molecular dynamics: Numerical methods and algorithmic techniques. *Parallel Computing* **2012**, *38*, 245—259, https://doi.org/10.1016/j.parco.2011.08.005.
36. Fedkin, M.V.; Shin, Y.K.; Dasgupta, N.; Yeon, J.; Zhang, W.; van Duin, D.; van Duin, A.C.T.; Mori, K.; Fujiwara, A.; Machida, M.; Nakamura, H.; Okumura, M. Development of the ReaxFF Methodology for Electrolyte–Water Systems. *J. Phys. Chem. A* **2019**, *123 (10)*, 2125–2141, https://doi.org/10.1021/acs.jpca.8b10453.
37. Valentina, I.; Haroutioun, A.; Fabrice, L.; Vincent, V.; Roberto, P. Poly (Lactic Acid)-Based Nanobiocomposites with Modulated Degradation Rates. *Materials* **2018**, *11, 1943*, https://doi.org/10.3390/ma11101943.
38. Gaussian 16, Revision A.03. Frisch, M. J.; Trucks, G. W.; Schlegel, H. B.; Scuseria, G. E.; Robb, M. A.; Cheeseman, J. R.; Scalmani, G.; Barone, V.; Petersson, G. A.; Nakatsuji, H.; Li M.; Caricato, X.; Marenich, A. V.; Bloino, J.; Janesko, B. G.; Gomperts, R.; Mennucci, B.; Hratchian, H. P.; Ortiz, J. V.; Izmaylov, A. F.; Sonnenberg, J. L.; Williams-Young, D.; Ding, F.; Lipparini, F.; Egidi, F.; Goings, J.; Peng, B.; Petrone, A.; Henderson, T.; Ranasinghe, D. ; Zakrzewski, V. G.; Gao, J.; Rega, N.; Zheng, G.; Liang, W.; Hada, M.; Ehara, M.; Toyota, K.; Fukuda, R.; Hasegawa, J.; Ishida, M.; Nakajima, T.; Honda, Y.; Kitao, O.; Nakai, H.; Vreven, T.; Throssell, K.; Montgomery, J. A., Jr.; Peralta, J. E.; Ogliaro, F.; Bearpark, M. J.; Heyd, J. J.; Brothers, E. N.; Kudin, K. N.; Staroverov, V. N.; Keith, T. A.; Kobayashi, R.; Normand, J.; Raghavachari, K.; Rendell, A. P.; Burant, J. C.; Iyengar, S. S.; Tomasi, J.; Cossi, M.; Millam, J. M.; Klene, M; Adamo, C.; Cammi, R.; Ochterski, J. W.; Martin. R. L.; Morokuma, K.; Farkas, O.; Foresman, J. B.; Fox, D. J. Gaussian, Inc., Wallingford CT, 2016.
39. Yildirim, E.; Dakshinamoorthy, D.; Peretic, M.J.; Pasquinelli, M.A.; Mathers, R.T. Synthetic Design of Polyester Electrolytes Guided by Hydrophobicity Calculations. *Macromolecules* **2016**, *49*, 7868–7876, https://doi.org/10.1021/acs.macromol.6b01452.
40. Gilardoni, F.; Weber, J.; Chermette, H.; Ward, T.R. Reactivity indices in density functional theory: A new evaluation of the condensed fukui function by numerical integration. *J. Phys. Chem. A* **1998**, *102*, 3607–3613.
41. Berger, G. Using conceptual density functional theory to rationalize regioselectivity: A case study on the nucleophilic ring-opening of activated aziridines. *Comput. Theor. Chem.* **2013**, *1010*, 11–18, https://doi.org/10.1016/j.comptc.2012.12.029.
42. Xu, L.; Crawford, K.; Gorman, C.B. Effects of Temperature and pH on the Degradation of Poly (lactic acid) Brushes. *Macromolecules* **2011**, *44*, 4777–4782, https://doi.org/10.1021/ma2000948.
43. Zhang, L.; Zhao, G.; Wang, G. Investigation on the α/δ Crystal Transition of Poly(L-lactic Acid) with Different Molecular Weights. *Polymers* **2021**, *13, 3280*, 1-16, https://doi.org/10.3390/polym13193280
44. Chu, C.C. Hydrolytic degradation of polyglycolic acid: Tensile strength and crystallinity study. *J. Appl. Polym. Sci.* **1981**, *26*, 1727–1734, https://doi.org/10.1002/app.1981.070260527.
45. de Jong, S.; Arias, E.; Rijkers, D.T.; van Nostrum, C.; Bosch, J.K.-V.D.; Hennink, W. New insights into the hydrolytic degradation of poly(lactic acid): Participation of the alcohol terminus. *Polymer* **2001**, *42*, 2795–2802, https://doi.org/10.1016/s0032-3861(00)00646-7.
